# Dermal Lipogenesis Inhibits Adiponectin Production in Human Dermal Fibroblasts while Exogenous Adiponectin Administration Prevents against UVA-Induced Dermal Matrix Degradation in Human Skin

**DOI:** 10.3390/ijms17071129

**Published:** 2016-07-14

**Authors:** Chien-Liang Fang, Ling-Hung Huang, Hung-Yueh Tsai, Hsin-I Chang

**Affiliations:** 1 Division of Plastic and Reconstructive Surgery, Department of Surgery, Ditmanson Medical Foundation Chia-Yi Christian Hospital, No. 539, Zhongxiao Rd., Chiayi City 60002, Taiwan; fangcl68@hotmail.com; 2 Department of Biochemical Science and Technology, National Chia Yi University, No. 300, Syuefu Rd., Chiayi City 60004, Taiwan; s102605@alumni.ncyu.edu.tw (L.-H.H.); s1040681@mail.ncyu.edu.tw (H.-Y.T.)

**Keywords:** adiponectin, human dermal fibroblast, photoaging, adipogenic differentiation medium, lipogenesis

## Abstract

Adiponectin is one of the most abundant adipokines from the subcutaneous fat, and regulates multiple activities through endocrine, paracrine, or autocrine mechanisms. However, its expression in adipogenic induced fibroblasts, and the potential role in photoaging has not been determined. Here, human dermal fibroblasts, Hs68, were presented as a cell model of dermal lipogenesis through stimulation of adipogenic differentiation medium (ADM). Similar to other studies in murine pre-adipocyte models (i.e., 3T3-L1), Hs68 fibroblasts showed a tendency to lipogenesis based on lipid accumulation, triglyceride formation, and the expressions of PPAR-γ, lipoprotein lipase (LPL), and FABP4 mRNA. As expected, ADM-treated fibroblasts displayed a reduction on adiponectin expression. Next, we emphasized the photoprotective effects of adiponectin against UVA-induced damage in Hs68 fibroblasts. UVA radiation can downregulate cell adhesion strength and elastic modulus of Hs68 fibroblasts. Moreover, UVA radiation could induce the mRNA expressions of epidermal growth factor receptor (EGFR), adiponectin receptor 1 (AdipoR1), matrix metalloproteinase-1 (MMP-1), MMP-3, and cyclooxygenase-2 (COX-2), but downregulate the mRNA expressions of type I and type III collagen. On the other hand, post-treatment of adiponectin can partially overcome UVA-induced reduction in the cell adhesion strength of Hs68 fibroblasts through the activation of AdipoR1 and the suppression of EGF-R. In addition, post-treatment of adiponectin indicated the increase of type III collagen and elastin mRNA expression and the decrease of MMP-1 and MMP-3 mRNA expression, but a limited degree of recovery of elastic modulus on UVA-irradiated Hs68 fibroblasts. Overall, these results suggest that dermal lipogenesis may inhibit the expression of adiponectin while exogenous adiponectin administration prevents against UVA-induced dermal matrix degradation in Hs68 fibroblasts.

## 1. Introduction

Subcutaneous adipose tissue lies just beneath the dermis of the skin. The increase of subcutaneous adipose tissue is speculated to induce dysfunctional changes in the dermis, decreasing dermal elasticity and result in the formation of sagging [1]. Sugihara et al. reconstructed skin system with rat skin cells and fat cells in a three-dimensional collagen gel matrix culture and reported that fat cells inhibited the proliferation of dermal fibroblasts [2]. Additionally, an adipogenesis-defect animal model indicated that intradermal adipocytes could mediate fibroblast recruitment during skin wound healing. Thus, it seems likely that subcutaneous adipocytes influence dermal condition through endocrine, paracrine, or autocrine mechanisms.

Subcutaneous adipose tissue is now considered as a dynamic organ which regulates the uptake, storage, and release of lipids and also secretes various bioactive substances, termed adipokines [3]. Adiponectin is one of the most abundant adipokines from the subcutaneous fat, and regulates multiple activities through endocrine, paracrine, or autocrine mechanisms. Generally, reduced adiponectin levels have been detected in patients with obesity, type II diabetes, and metabolic syndrome and, hence, adiponectin appears to be an insulin-sensitizing, anti-inflammatory, and anti-apoptotic mediator [4]. In in vitro studies, adiponectin was confirmed to reduce IL-6 expression in normal human keratinocytes and increase production of hyaluronic acid (HA) and collagen in dermal fibroblasts. Moreover, adiponectin could reduce IL-17 synthesis through the suppression of adiponectin receptor 1 (AdipoR1) in murine dermal γδ-T cells to regulate skin inflammation [5]. Therefore, we hypothesized in the present work that adiponectin may play a protective role against UV-induced dermal matrix degradation (ECM).

Ultraviolet (UV) radiation is considered a cause of skin aging and may result in pathological changes in the skin. Particularly, UVA, a long-known aging ray and a significant source of oxidative stress (ROS), plays a major role in the photoaging of human skin [6,7]. Photoaged skin typically shows the loss of elasticity, slowing of skin cell growth associated with slower wound healing and may, thus, become thin and vulnerable [8]. As we know, excessive exposure to UV irradiation induces damage to the dermal connective tissue and which leads to a large of inflammatory responses, including TNF-α, IL-1α, and a stress response gene cyclooxygenase-2 (COX-2) [9]. Upon stimulation by the inflammatory responses, the degradation of dermal ECM is being processed through activation of matrix metalloproteinases (MMP), and the decrease of collagen production [10]. Of importance, adiponectin exerts anti-inflammatory activity via inhibition of TNF-α, IL-6, IL-8, VCAM-1, and ROS production on a number of cell types [11]. However, there is a little information available concerning the impact of the adiponectin on UVA-damaged skin and its mechanism of regulation. To this end, this study will focus on the adiponectin expression under lipogenic conditions and then evaluate photo protective effects of adiponectin on UVA-induced damage in Hs68 human dermal fibroblasts.

## 2. Results

### 2.1. The Effect of ADM on Cell Viability of Hs68 Fibroblasts

**Figure 1:**
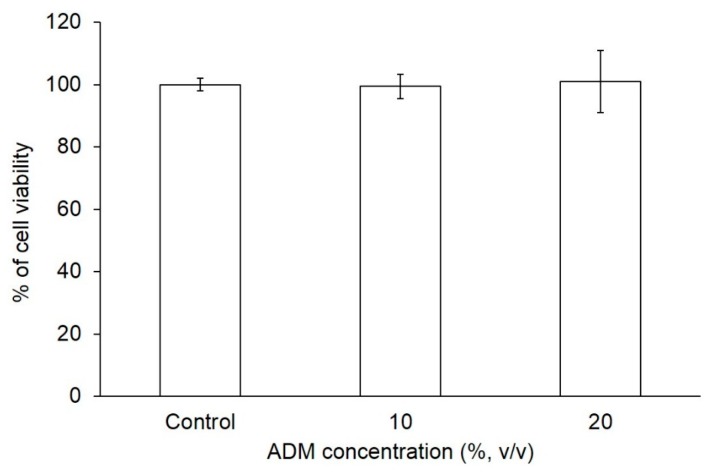
The effect of ADM concentration on cell viability. Human dermal fibroblasts, Hs68 were incubated with ADM for seven days. Data are expressed by the mean of percent cell viability compared to control ± standard deviation, *n* = 3.

3T3-L1 fibroblasts are the most commonly studied adipogenic cell line. Generally, 3T3-L1 fibroblasts undergo adipogenic differentiation rapidly, within one week in most instances. In contrast to human bone marrow mesenchymal stroma cells (hMSCs) and 3T3-L1 fibroblasts, little is known about the adipogenesis of human skin fibroblasts [12]. To determine the effect of adipogenic differentiation medium (ADM) on proliferation of Hs68 fibroblasts, cell viability was measured using MTT (3-(4,5-dimethylthiazol-2-yl)-2,5-diphenyltetrazolium bromide) tetrazolium reduction assay. In comparison with control, Hs68 fibroblasts were incubated with ADM at concentrations of 10% and 20% for seven days to induce adipogenic differentiation. As shown in Figure 1, there is no significant difference in the cell proliferation between control and ADM treatment groups.

### 2.2. ADM Stimulated Lipid Accumulation in Hs68 Fibroblasts

**Figure 2:**
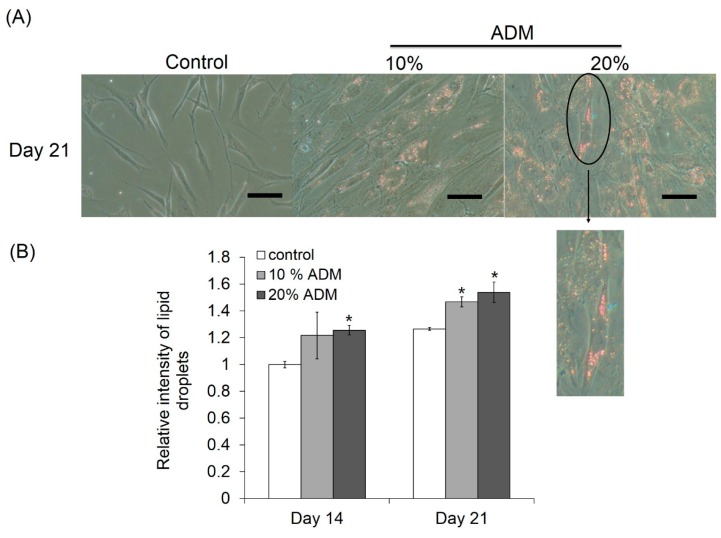
Lipid accumulation in human dermal fibroblasts, Hs68. (**A**) The cells were stained with Oil Red O solution after 21 days incubation with ADM to determine lipid droplet accumulation. (×200 magnification, Scale bar = 100 μm); and (**B**) quantification of Oil Red O staining has showed that there is no apparent trend of O.D. value with ADM treatment. The results were expressed by the mean absorbance of Oil Red O residues dissolved in isopropanol compared to control ±standard deviation, *n* = 3. * *p* < 0.05 with respect to control.

**Figure 3:**
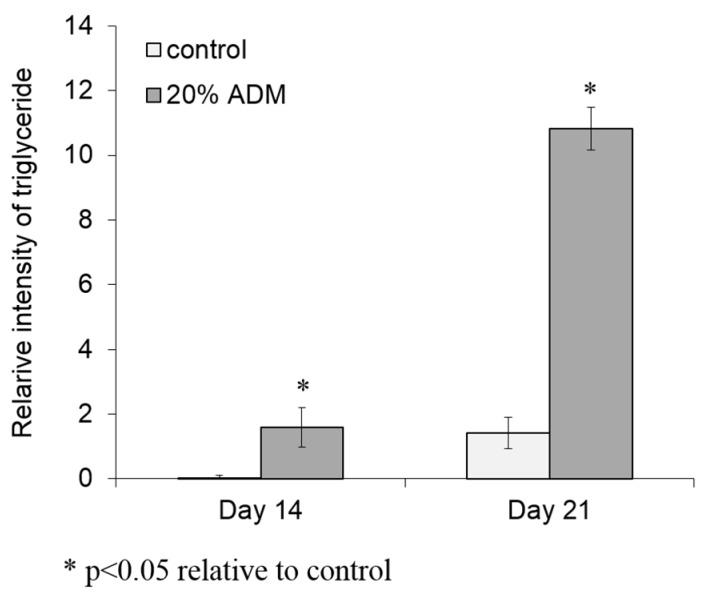
ADM increased triglyceride accumulation in human dermal fibroblasts, Hs68. Hs68 fibroblasts were treated with 20% ADM. After 14 and 21 days, the cellular TG contents were measured with TG adipogenesis kit at 570 nm. The results were expressed by the mean intensity of triglyceride compared to control ± standard deviation, *n* = 3.

To prove the possibility of lipid accumulation in dermal tissue, Hs68 fibroblasts were incubated with different concentrations of ADM for 14 and 21 days to investigate the level of lipogenesis. During adipogenic differentiation, cells should exhibit very close morphological appearances, characteristic of adipocytes with the cytoplasm accumulating growing lipid droplets, which can be visualized by Oil Red O staining. After 21 days incubation with ADM, cell shape changes, and the increase in lipid droplets were observed in Hs68 fibroblasts (Figure 2A). Additionally, the lipid accumulation in Hs68 fibroblasts was increased with ADM concentration and incubation time (Figure 2B). Next, we were interested to determine the ADM-mediated effect on triglyceride formation. Similar to lipid accumulation, Hs68 fibroblasts also showed higher triglyceride formation than control after 21 days of incubation with ADM (Figure 3). Taken together, these results suggest that ADM can dose-dependently induce lipid accumulation in Hs68 fibroblasts.

### 2.3. Expression of Adipogenesis Markers in ADM-Induced Hs68 Fibroblasts

**Figure 4:**
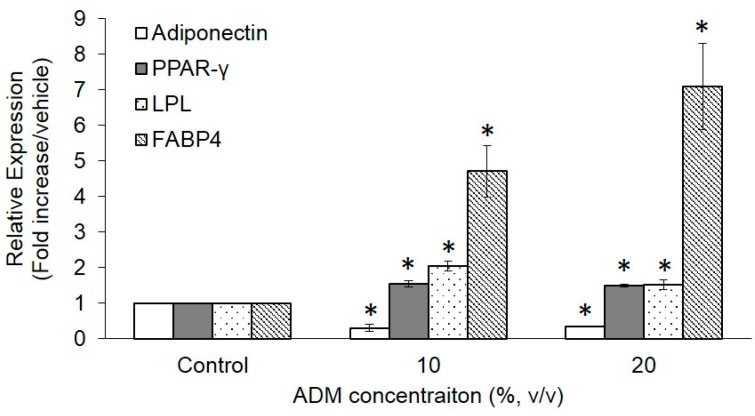
Effect of ADM concentrations on adipogenic marker genes in human dermal fibroblasts, Hs68. Hs68 fibroblasts were induced to undergo the differentiation process in the absence or presence of ADM for three days. RNA samples were prepared from cells at the indicated time point after treatment. Real-time RT-PCR analysis was performed to examine gene expression of four adipogenesis markers (relative to GADPH). The data were presented as the means ± standard deviation, *n* = 3–6. * *p* < 0.05 with respect to control.

**Figure A1:**
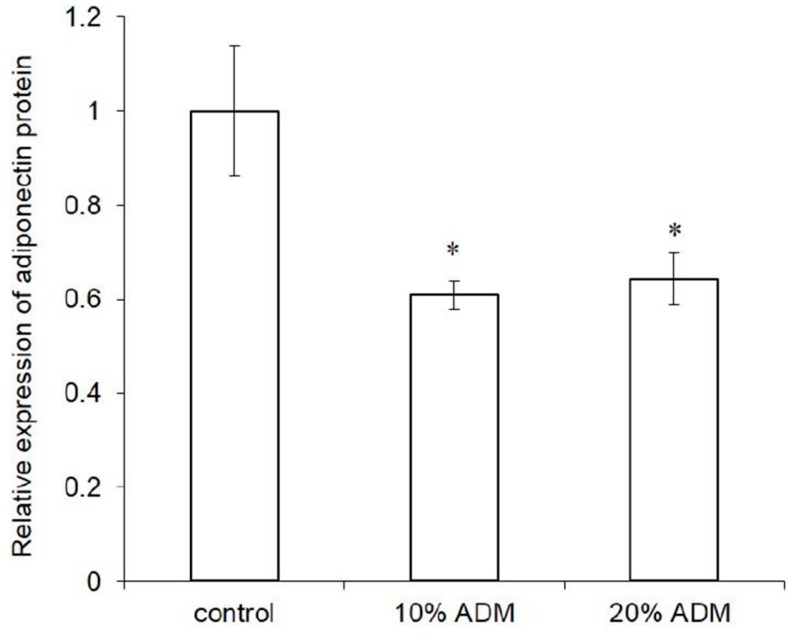
Adiponectin expression in Hs68 fibroblasts with or without ADM treatment. Hs68 fibroblasts were incubated for 7 days with or without ADM treatment (10% or 20%). A comparison of adiponectin protein content were determined by ELISA and expresses as relative adiponectin quantity. Data are expressed by the mean intensity of adiponectin expression compared to control ± standard deviation, *n* = 3. * *p* = 0.05.

Adipogenesis is a multi-step process involving a cascade of transcription factors and cell-cycle proteins regulating gene expression and leading to adipogenic differentiation. To characterize the mechanism of ADM-induced lipid accumulation in Hs68 fibroblasts, we measured the adipogenic differentiation gene expression such as adiponectin, peroxisome proliferator-activated receptor γ (PPAR-γ), lipoprotein lipase (LPL), and fatty acid binding protein 4 (FABP4). Figure 4 demonstrated that three days of ADM treatment stimulated PPAR-γ, LPL, and FABP4 mRNA expression with respect to basal expression but suppressed adiponectin mRNA expression. We also checked the expression pattern of adiponectin protein in ADM-induced Hs68 fibroblasts and compared it with the expression in control. As expected, the expression pattern of adiponectin protein was similar to that of mRNA in ADM-induced Hs68 fibroblasts (*p* < 0.05, Figure A1). Consequently, adiponectin was chosen to study the impact on UVA-irradiated Hs68 fibroblasts.

### 2.4. Adiponectin Can Increase Cell Adhesion Strength of UVA-Irradiated Hs68 Fibroblasts

**Figure 5:**
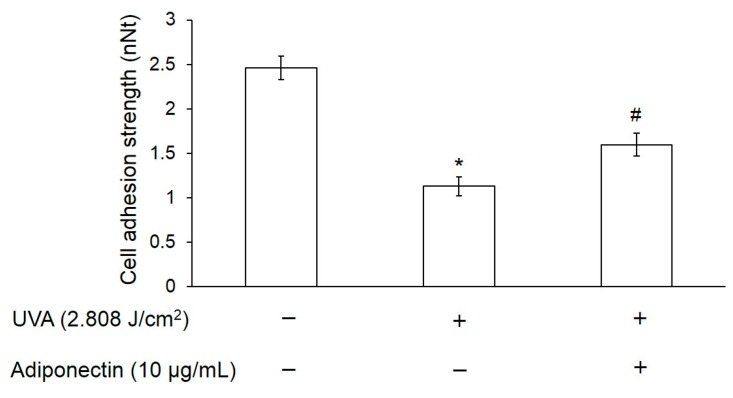
The effect of UVA radiation and adiponectin on cell adhesion strength of human dermal fibroblasts, Hs68. The cell adhesion strength of Hs68 fibroblasts was reduced after 4 h of UVA radiation but partially recovered with 12 h treatment of adiponectin. Data were expressed by the mean of cell adhesion strength compared to control ± standard deviation, *n* = 30. * *p* < 0.05 with respect to control; # *p* < 0.05 with respect to cells treated with UVA radiation. (The symbols of – and + are represented as the absence and presence of UVA or adiponectin.)

Cell adhesive ability is an important phenomenon that controls the behavior of cells, such as their morphology, migration, growth, and differentiation. To study the effect of UVA irradiation on cell adhesion ability, we used dielectrophoresis (DEP) force to measure the cell adhesion strength of Hs68 fibroblasts. Hs68 fibroblasts were exposed to UVA radiation at a dose of 2.808 J/cm^2^. Cells were cultured in DMEM with and without adiponectin (10 μg/mL) for 12 h after exposure to UVA radiation. Figure 5 revealed that cell adhesion strength of Hs68 fibroblasts with exposure to UVA radiation was 2.2-fold lower than control and hence UVA radiation presented a dominant negative effect on cell adhesion strength. In contrast, UVA-irradiated Hs68 fibroblasts can increase cell adhesion force by 42% through post-treatment with adiponectin. Therefore, UVA radiation could reduce the cell adhesion ability of Hs68 fibroblasts but post-treatment with adiponectin can minimize the adverse effect to the cells.

### 2.5. Adiponectin Can Increase Elastic Modulus of UVA-Irradiated Hs68 Fibroblasts

**Figure 6:**
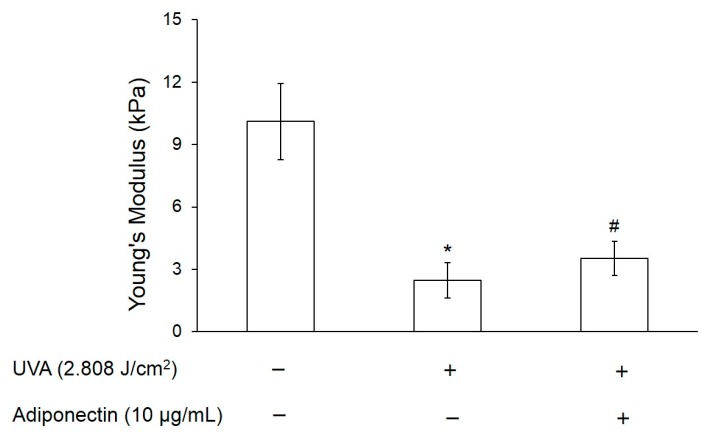
The effect of UVA radiation and adiponectin on the Young’s modulus of human dermal fibroblasts, Hs68. Young’s modulus was measured by AFM. Data were expressed by the mean of young’s modulus compared to control ±standard deviation, *n* = 25. * *p* < 0.05 with respect to control; # *p* < 0.05 with respect to cells treated with UVA radiation.

Skin wrinkle can cause the loss of elasticity and, hence, we examined the effect of adiponectin on elastic modulus of UVA-irradiated Hs68 fibroblasts by Atomic-force microscopy (AFM). Figure 6 exhibited that the Young’s modulus of hs68 fibroblasts was declined from 10.11 to 2.46 kPa after exposure to the UVA radiation. This indicated that UVA radiation could cause a significant damage on cell elasticity. However, UVA-irradiated Hs68 fibroblasts can increase the Young’s modulus by 44% through post-treatment with adiponectin. Like the results presented in cell adhesion strength, post-treatment with adiponectin could minimize the reduction of cell elastic modulus. 

### 2.6. The Effect of Adiponectin on Gene and Protein Expression of UVA-Irradiated Human Dermal Fibroblasts

**Figure 7:**
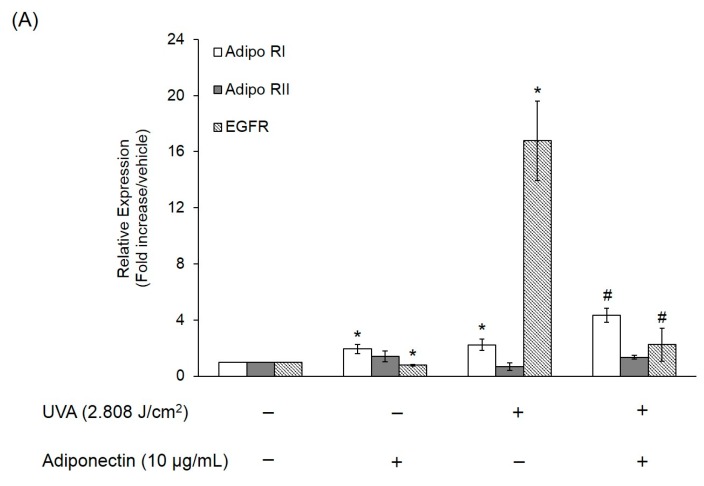

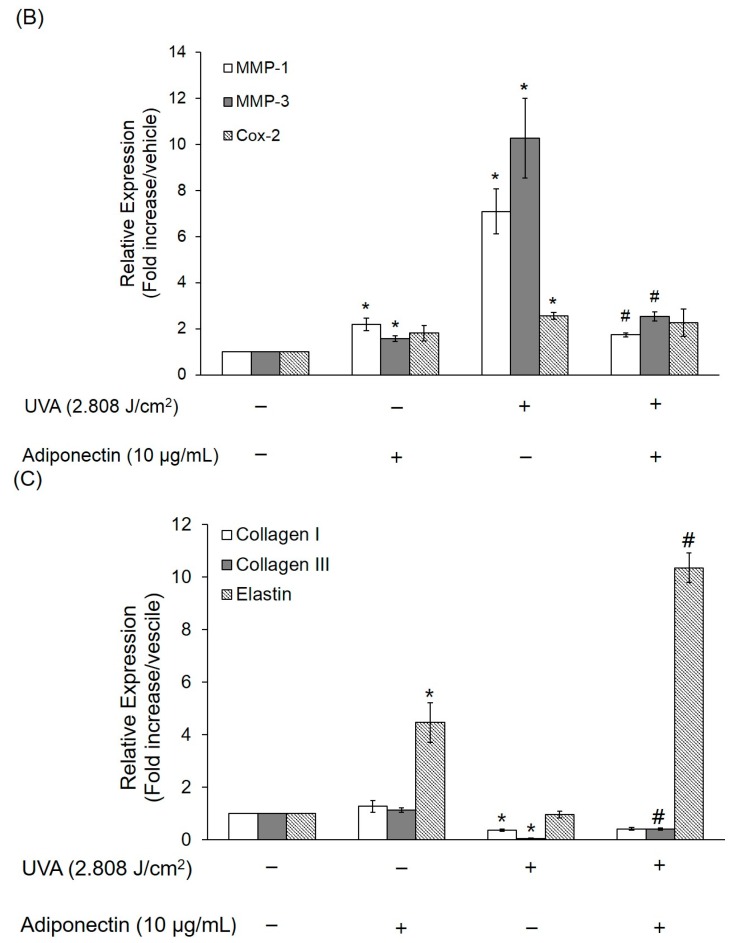
The effect of adiponectin on gene expression of UVA-irradiated Hs68 fibroblasts. (**A**) Gene expression of AdipoR1, AdipoR2, and EGFR on Hs68 fibroblasts, were measured by real-time PCR; (**B**) levels of MMP-1, MMP-3, and COX-2 mRNA expression of Hs68 fibroblasts; and (**C**) levels of elastin, type I and III collagen mRNA expression of Hs68 fibroblasts. The data were presented as the means ± standard deviation, *n* = 3–6. * *p* < 0.05 with respect to control; # *p* < 0.05 with respect to UVA.

**Figure 8:**
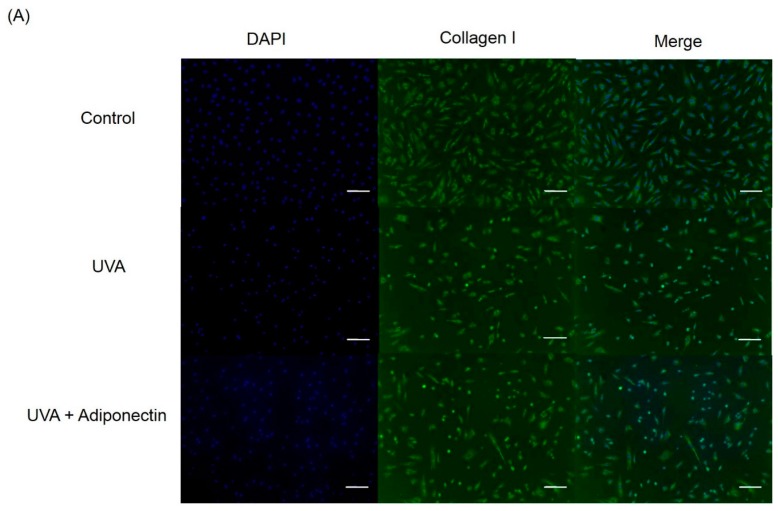

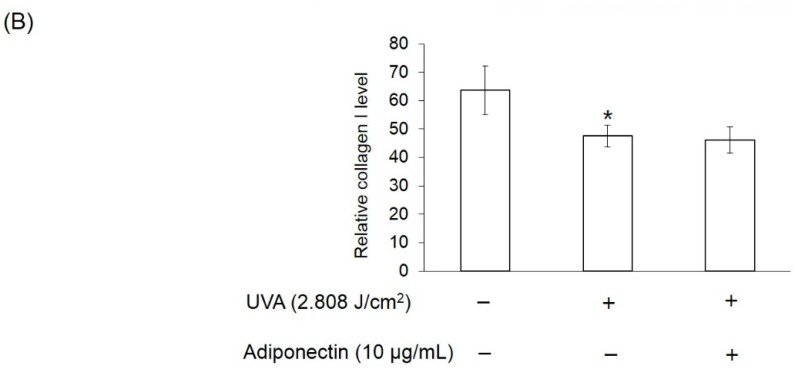
The effect of adiponectin on type I collagen expression in UVA-irradiated Hs68 fibroblasts. (**A**) Immunofluorescence staining of Hs68 fibroblasts using specific type I collagen antibody (×100 magnification, Scale bar = 200 μm); and (**B**) quantification of immunofluorescence staining of Hs68 fibroblasts. Green fluorescence staining indicates the presence of type I collagen. Blue staining illustrates the DAPI nuclei counterstain. * *p* < 0.05 with respect to control.

**Figure 9:**
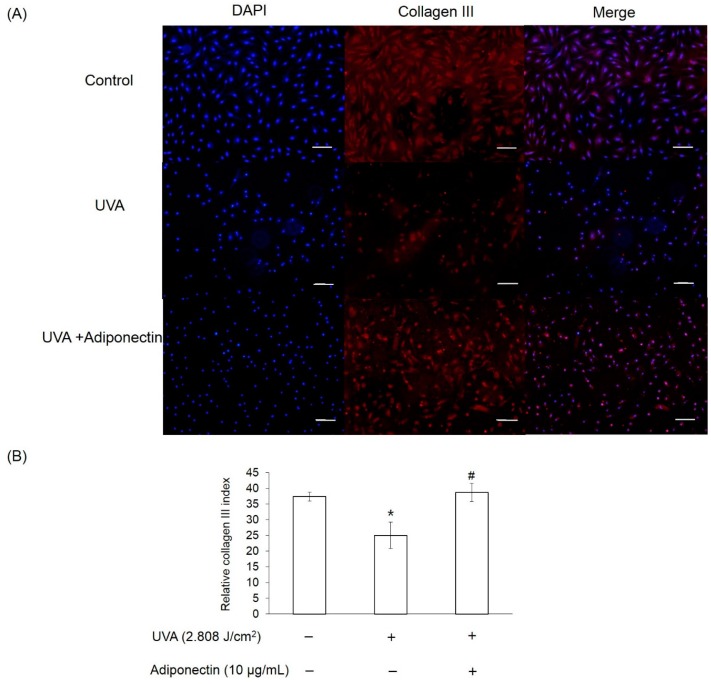
The effect of adiponectin on type III collagen expression in UVA-irradiated Hs68 fibroblasts. (**A**) Immunofluorescence staining of Hs68 fibroblasts using specific type III collagen antibody (×100 magnification, Scale bar = 200 μm); and (**B**) quantification of immunofluorescence staining of Hs68 fibroblasts. Red fluorescence staining indicates the presence of type III collagen. Blue staining illustrates the DAPI nuclei counterstain. * *p* < 0.05 with respect to control; # *p* < 0.05 with respect to UVA.

**Figure 10:**
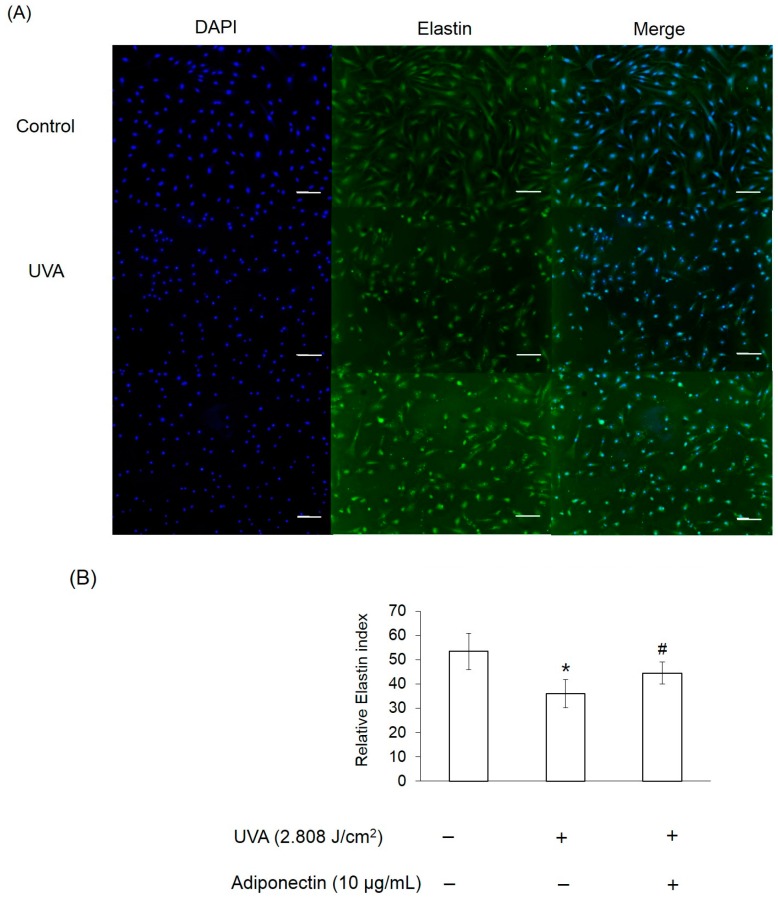
The effect of adiponectin on elastin expression in UVA-irradiated Hs68 fibroblasts. (**A**) Immunofluorescence staining of Hs68 fibroblasts using specific elastin antibody (green fluorescence, ×100 magnification, Scale bar = 200 μm); and (**B**) quantification of immunofluorescence staining of Hs68 fibroblasts. Green fluorescence staining indicates the presence of elastin. Blue staining illustrates the DAPI nuclei counterstain. * *p* < 0.05 with respect to control; # *p* < 0.05 with respect to UVA.

We next examined the mechanism by which adiponectin modulates adhesion strength and elasticity of Hs68 fibroblasts after exposure to UVA-irradiation. Total RNA was extracted from Hs68 fibroblasts after exposure to UVA irradiation (2.808 J/cm^2^) and adiponectin treatment (10 μg/mL). First, we investigated expression levels of adiponectin and epidermal growth factor receptors (EGFR). Adiponectin receptors, AdipoR1 and AdipoR2, serve as receptors for adiponectin and mediate increased AMP-activated kinase (AMPK) and PPARs activities, thereby regulating glucose and lipid metabolism [13]. In this study, we examined whether the expressions of AdipoR1 and AdipoR2 in Hs68 fibroblasts were regulated by UVA-irradiation. In comparison with control, UVA radiation stimulated AdipoR1 expression, but not AdipoR2 (Figure 7A). On the other hand, EGFR is involved in the UV signal transduction pathway leading to skin inflammation, photoaging, and cancer development [14]. Similar to other research, we assessed that UVA irradiation can induce EGFR expression in Hs68 fibroblasts. As expected, post-treatment of adiponectin in Hs68 fibroblasts can suppress UVA-induced EGFR expression and stimulate AdipoR1 expression. Liao et al. demonstrated that the expression of AdipoR1 was substantially greater than AdipoR2, and adiponectin can elicit antihypertrophic action via the inhibition of heparin-binding EGF-mediated EGFR activation [15]. Then, we measured the expression of genes involved in the inflammation and extracellular matrix degradation. MMPs are thought to be responsible for matrix turnover and degradation. Additionally, MMPs also represent a marker of inflammation since they regulate inflammation and immunity. UVA irradiation induced a seven-fold increase in the mRNA level of MMP-1, a 10-fold increase in the mRNA level of MMP-3, and a 2.5-fold increase in the mRNA level of COX-2 relative to levels in controls (Figure 7B). Post-treatment of Hs68 fibroblasts with adiponectin can significantly suppress MMP-1 and MMP-3 expressions but had a minor inhibitory effect on COX-2 expression. Fisher et al. have reported that UV irradiation reduced type I and III procollagen mRNA and protein levels in human skin in vivo [16]. In here, we also presented that UVA radiation inhibited the gene expressions of type I and III procollagen but not elastin (Figure 7C). Of the note, adiponectin can increase gene expressions of elastin and type III procollagen but show no effect on type I procollagen. This increase was also shown by immunofluorescence staining in adiponectin-treated Hs68 fibroblasts compared to untreated cells after exposure to UVA irradiation (Figure 8, Figure 9 and Figure 10).

## 3. Discussion

Rakar et al. have demonstrated that primary human dermal fibroblasts could be induced to differentiate towards adipocytes, osteoblasts, and chondrocytes using different induction media [17]. Based on the increase in lipid accumulation and triglyceride formation, we can approximately define that Hs68 fibroblasts were successfully induced to lipogenesis by ADM stimulation (Figure 2 and Figure 3). The induction of lipogenesis can be further described by gene expression of PPAR-γ, LPL, and FABP4. PPAR-γ has appeared as the key determinants of terminal adipocyte differentiation [18]. LPL catalyzes the hydrolysis of triglyceride while it is rich in adipose tissue. The expression of LPL mRNA has often been considered as an early marker of adipocyte differentiation [19]. Additionally, FABP4, commonly known as adipocyte protein 2 (aP2), is a carrier protein for fatty acids that is primarily expressed in adipocytes and macrophages [20]. As expected, PPAR-γ, LPL, and FABP4 mRNA expression level were increased but adiponectin mRNA expression level was reduced with ADM treatment (Figure 4). Adiponectin is one of the most abundant adipokines from the subcutaneous fat, and its level in skin tissue is significantly correlated with the mass of subcutaneous fat tissue. In humans, plasma adiponectin concentrations fall with increasing obesity, and this effect is greater in men than in women [21]. Recently, Shibata studies mentioned that wound closure and keratinocyte re-epithelialization was significantly delayed in adiponectin-deficient mice compared with wild-type mice [22]. Thus, adiponectin has beneficial and direct effects on cutaneous wound healing, especially in diabetic patients whose adiponectin levels are constitutively decreased. 

Cell adhesion is a critical factor that is associated with many aspects of cell behavior. The adhesive interactions between cell membrane receptors and ECM ligands are known to affect cell growth, differentiation, and migration [23]. Our results demonstrated that UVA irradiation can reduce cell adhesion strength and that may be due to cell senescence. Typically, dermal fibroblasts undergo senescence rather than apoptosis after DNA damage or multiple rounds of cell division since the presence of senescent fibroblasts in the dermis of photoaged skin has been shown by β-galactosidase staining [24]. A recent study indicated an age-dependent decline in the expression of E-cadherin, the cell-cell adhesion molecule, in the germline stem cells of the *Drosophila* ovary and testis [25]. Similarly, the adhesion strength between hematopoietic stem cells (HSC) appears to decrease with age, as the ability to mobilize HSCs drastically increases in older mice [26]. This is the first time, to our knowledge, that UVA irradiation has been reported to reduce cell adhesion strength of Hs68 fibroblasts and these findings support an interpretation of photoaging as an acceleration of cellular senescence (Figure 5).

Changes in cell elasticity have been implicated in the pathogenesis of many human diseases including vascular disorders, arthritis and cancer. Li et al. have displayed that MCF-7 breast cancer cells had Young’s modulus 1.4–1.8 times lower than normal breast epithelial cells (MCF-10A) [27]. Moreover, Hsieh et al. used AFM to demonstrate that the elastic properties of normal chondrocytes was about 2.8-fold higher than OA chondrocytes [28]. Similar to the researches of Li et al. and Hsieh et al., we demonstrated that UVA irradiation can cause cell damage and result in reducing cell elasticity (Figure 6). Collagen is a keystone of skin formation and repair, playing a role keeping skin tensility and elasticity [29]. According to previous studies, skin exposed to UV irradiation can induce photoaging through upregulation of MMPs and consecutive ECM breakdown [30]. Our results indicated that the exposure of Hs68 fibroblasts to UVA radiation down-regulated type I and III collagen gene expression and that suppressed ECM synthesis (Figure 7C). Additionally, the exposure of Hs68 fibroblasts to UVA radiation induced MMP-1, MMP-3, and COX-2 mRNA expression and that that can induce ECM degradation (Figure 7B). Therefore, our study suggested that UVA irradiation can reduce skin elasticity through inhibition of collagen synthesis and MMP activation.

Conversely, adiponectin could minimize the suppression effect on cell elastic modulus and adhesion strength of UVA-irradiated Hs68 fibroblasts. Adiponectin exerts its function through its receptors AdipoR1 and AdipoR2. AdipoR1 is one type of adiponectin receptor found in many tissues, especially in skeletal muscle, whereas AdipoR2 is commonly found in the liver [31]. Consistent with our results, Shibata et al. demonstrated that adiponectin upregulated gene expression of AdipoR1 but not AdipoR2 in human keratinocytes [22]. In the present study, UVA radiation induced AdipoR1 and EGFR mRNA expression on Hs68 fibroblasts (Figure 7A) whereas Kim et al. demonstrated that UV irradiation (UVA + UVB) on human skin can reduce the gene expressions of adipoR1 and adipoR2 in dermal and subcutaneous fat tissues [32]. One possible explanation for the discrepancy between our findings and the work of Kim et al. are differences in experimental conditions, such as the presence or absence of UVB and the nature of the samples*.* Alternatively, EGFR regulates cell growth, survival, adhesion, and migration in fibroblasts [33]. Similar to our findings, Liao et al. revealed that adiponectin inhibited the phosphorylation of the epidermal growth factor (EGF) in cardiomyocytes [15]. Apart from that, inhibition of the EGFR is able to induce the formation of desmosomes and promote cell adhesion in squamous cell cancer cells [34]. Maheshwari et al. have previously shown that addition of soluble EGF to NR6 fibroblasts, which lack EGFR, results in a sustained decrease in adhesion to fibronectin [35]. Moreover, EGF-treated NR6 fibroblasts can stimulate EGFR activation leading to the loss of focal adhesions and a concomitant reduction in cell adhesion strength. Taken these findings together, UVA radiation could reduce cell adhesion ability of Hs68 fibroblasts but post-treatment with adiponectin can minimize the adverse effect to the cells through the upregulation of AdipoR1 expression and the downregulation of EGFR expression. 

Since anti-inflammatory properties of adiponectin have been well-investigated and discussed, we expected that adiponectin can inhibit UVA-induced MMP activation. Interestingly, adiponectin can induce MMP-1 and MMP-3 expression of Hs68 fibroblasts in normal condition and that match the enhanced effects of adiponectin on the production of NO, IL-6, MMP-1, and MMP-3 in OA cartilage and in primary chondrocytes [36]. Therefore, adiponectin may stimulate MMP-1 and MMP-3 expression in normal conditions, but suppress UVA-induced expression levels. Apart from that, post-treatment of adiponectin did not suppress UVA-induced COX-2 expression. On the other hand, our results revealed that adiponectin promoted gene and protein expression of collagen type III and elastin in Hs68 fibroblasts in the presence or absence of UVA irradiation (Figure 7C, Figure 9 and Figure 10). Early studies demonstrated that direct stimulation of primary cardiac fibroblasts with adiponectin increased immunofluorescence detection of intracellular collagens type I and III [37]. Although only one animal study in abdominal aortic aneurysms presented that elastin was more preserved in adiponectin-administrated mice in comparison with control, our data provides new evidence that adiponectin also has stimulatory effect on elastin synthesis in the presence or absence of UVA irradiation [38]. Thus, post-treatment with adiponectin could minimize the reduction of cell elastic modulus through the downregulation of MMP expression and the upregulation of collagen and elastin expression.

In conclusion, our findings suggest that lipogenesis in Hs68 fibroblasts may reduce the expression of adiponectin; whereas exogenous adiponectin administration prevent against UVA-irradiated dermal damage in Hs68 fibroblasts through upregulation of collagen and elastin synthesis and downregulation of MMP expressions. 

## 4. Materials and Methods

### 4.1. Cell Culture and Reagents

Recombinant human adiponectin was purchased from PROSPEC (10 μg, CYT-434, Ness-Ziona, Israel) and added to 1 mL of DMEM supplemented with 10% *v*/*v* FBS, 100 units/mL penicillin, and 100 μg/mL of streptomycin to prepare a working solution of approximately 10 μg/mL. Adipogenesis differentiation medium (ADM) and all cell culture materials were purchased from Gibco (Grand Island, NY, USA). All solvents were of analytical reagent grade (J.T. Baker, Center Valley, PA, USA). Human foreskin fibroblasts (Hs68) were obtained from ATCC (Manassas, VA, USA). Hs68 fibroblasts were cultured in DMEM supplemented with 10% *v*/*v* FBS, 100 units/mL penicillin and 100 μg/mL of streptomycin. Cells were maintained at 37 °C with 5% CO_2_ in a humidified incubator. 

### 4.2. UVA Irradiation and Treatment

Human foreskin fibroblasts, Hs68, were seeded at a density of 5 × 10^5^ cells/6 cm dish and cultured in serum-free DMEM medium without phenol red to 70% confluence. Supplemental UVA radiation was supplied by a filtered lamp (365 nm, Vilber, Eberhardzell, Germany) suspended above the cells. The distance between the light source and cells was 19.5 cm. The UVA output measured by a UV radio meter (LT LUTRON, Taipei, Taiwan) was 0.195 mJ/cm^2^·s. The UVA irradiation was performed at 2.808 J/cm^2^. UVA dose (mJ/cm^2^) = Intensity (mW/cm^2^) × Time (s). Control cells were kept in the same culture condition without UVA exposure. After exposure to UVA irradiation, cells were washed with PBS and incubated with and without adiponectin (10 μg/mL) in serum-free DMEM for 12 h.

### 4.3. Cell Viability

Human foreskin fibroblasts, Hs68, were seeded at a density of 2 × 10^4^ cells/well in 12-well plates for cell survival studies. Cell medium was replaced with ADM every two days. After seven days, the supernatant was removed and 1 mL of MTT reagent (3-(4,5-Dimethylthiazol-2-yl)-2,5-diphenyltetrazolium bromide, 0.1 mg/mL) was added to each well to incubate at 37 °C for 4 h. The supernatant was removed and 1 mL dimethyl sulfoxide (DMSO) was added to each well and shaken for 15 min to dissolve the formazan. The absorbance was measured at 570 nm using the ELISA reader (Infinite M200, Tecan, Männedorf, Switzerland).

### 4.4. Oil Red O Staining

For adipogenic induction, cells were cultured for 14 and 21 days. Human foreskin fibroblasts, Hs68, were seeded at a density of 4.5 × 10^4^ cells/well in six-well plates. Cell medium was replaced with ADM every two days. After 14 and 21 days, the supernatant were removed and washed with PBS. For histochemical examination, adipogenic cultures were stained for the presence of intracellular lipid droplets using Oil Red O staining as an indicator of intracellular lipid accumulation. Oil red O staining was performed using the Oil Red O staining kit (Lifeline Cell Technology, Carlsbad, CA, USA). Briefly, cells were fixed for 30 min in 4% paraformaldehyde, washed with 100% 1,2-propanediol dehydration solution and then stained for 30 min at 37 °C with 0.5% Oil Red O solution. After removing Oil Red O solution, the images of cell morphology were taken by microscope (Eclipse Ti-E, Nikon Corporation, Shinagawa-ku, Tokyo, Japan) and CCD camera system (SPOT RT3*,* Diagnostic instruments Inc., Sterling Heights, MI, USA). Finally, 0.5 mL of 85% 1,2-propanediol stain differential solution was added to each well and the absorbance was measured at 500 nm using the ELISA reader (Infinite M200, Tecan).

### 4.5. Triglyceride Assay

The cellular contents of triglyceride in human dermal fibroblasts, Hs68 were determined using a TG adipogenesis assay kit (Sigma-Aldrich, St. Louis, MO, USA) according to the manufacturer’s instructions. Briefly, Hs68 fibroblasts were cultured at 5 × 10^4^ cells/well. Cell medium was replaced with ADM every two days. After 14 and 21 days of incubation, the supernatant were removed and washed with PBS. Lipid extraction buffer was added to each well and incubated at 90 °C for 30 min. After cooling the samples to room temperature, lipid extracts were transferred to 96-well plates and filled up with adipogenesis assay buffer. To degrade triglyceride to glycerol and fatty acids, each sample was added with lipase solution and placed at room temperature for 10 min. After adding master reaction mix to each well, the reaction was developed at 37 °C for 30 min and then absorbance was measured at 570 nm using the ELISA reader (Infinite M200, Tecan). Triglyceride content in cells was calculated from the triglyceride (triolein)-equivalent standard curve.

### 4.6. Measurement of Cell Adhesion Strength

**Figure 11:**
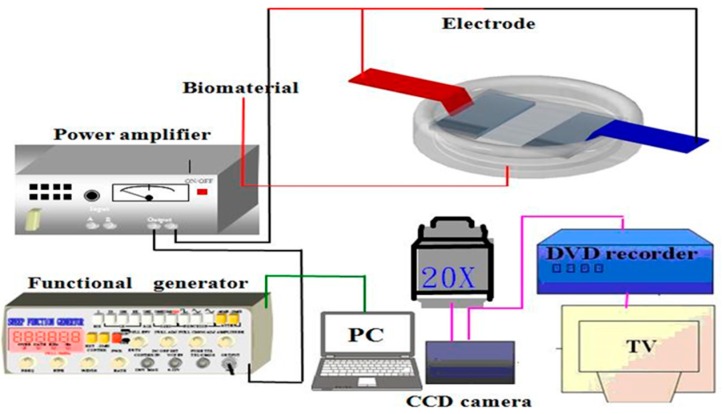
A schematic view of the dielectrophoresis method.

According to our previous study, cell adhesion strength was measured by dielectrophoresis [39]. Figure 11 shows the dielectrophoresis sensing system including an microscope (Model CK30, Olympus, Hatagaya, Shibuya-ku, Tokyo, Japan), a CCD camera (Model E-330, Olympus, Japan), a function generator (Model 33220A, Agilent, Santa Clara, CA, USA), a power amplifier (Model HSA4012, NF Corporation, Kohoku-ku, Yokohama, Japan), and palladium (Pd) electrodes. For the measurement of cell adhesion strength, Hs68 fibroblasts were seeded at a density of 3 × 10^5^ cells/6 cm dish*.* After exposure to UVA irradiation and post-treatment of adiponectin, the cell culture dishes were covered with the electrode plate. The cell adhesion strength was determined from cell detachment when an external electrical field was applied to cause dielectrophoresis. The external AC electrical field (1 MHz) was set at 0 V at the start of the measurement period and increased steadily until the cells moved away from the seeding position. Cell detachment was observed on the microscope and the images of the cells were recorded through a CCD camera connected to a computer. Later, the cell radii and spreading area were analyzed by ImageJ software (U. S. National Institutes of Health, Bethesda, MD, USA). Cell adhesion strength was converted from the electrical potential at which cell detachment occurred in accordance with the published procedure by Ay et al. Thirty cells were individually tested at each time point in triplicate.

### 4.7. The Young’s Modulus Analysis

The Young’s modulus of Hs68 fibroblasts was recorded with an atomic force microscope (AFM; Nanowizard™ AFM, JPK, FRG) as reported by Lien et al. [40]. The measurements were carried out using glasses as standard materials, whose Young’s modulus was determined from the load-displacement curves. Hs68 fibroblasts were seeded at a density of 2 × 10^5^ cells/6 cm dish for studying the effect of adiponectin on UVA-irradiated cells*.* After UVA-irradiation and post-treatment of adiponectin, cells were brought to the AFM and allowed to equilibrate to ambient conditions for 10 min (~25 °C) in DMEM with 10% FBS. Ten cells were typically selected per UV irradiation condition in each experiment, with a total of 30 cells, from three individual experiments.

### 4.8. Quantitative Real-Time PCR

Human foreskin fibroblasts, Hs68, were seeded at a density of 8 × 10^4^ cells/6 cm dish under adipogenic induction for seven days or 5 × 10^5^ cells/6 cm dish for the study of UVA radiation exposure. For adipogenic induction study, cells were cultured under adipogenesis differentiation medium for seven days and then total RNA was extracted from the Hs68 fibroblasts using REzol^TM^ C&T reagent (Protech Technologies, Taipei, Taiwan) according to the manufacturer’s instructions. For the photoaging study, total RNA was extracted from Hs68 fibroblasts after exposure to UVA irradiation (4 h) and post-treatment of adiponectin (12 h). RNA (1 μg) was reverse transcribed using TProfessional basic (Biometra, Göttingen, Germany). The resulting cDNA (equivalent to 20 ng) was used in a StepOnePlus™ Real-Time PCR System with a FastStart DNA Master-PLUS SYBR Green I (Applied Biosystems, Foster City, CA, USA). The primers sequences were listed in Table 1. Each sample was corrected using the mean cycle threshold (*C*_t_) value for GADPH. Relative gene expression was analyzed using the Δ*C*_t_ method and expressed as fold change (2^−ΔΔ*C*t^) T relative to the expression values in non-stimulated cells.

### 4.9. Immunofluorescence Staining

Hs68 fibroblasts were seeded at a density of 2 × 10^5^ cells/6 cm dish. After exposure to UVA radiation, cells were cultured in DMEM with or without adiponectin (10 μg/mL) for 24 h. After removing the supernatant, cells were fixed with 10% formaldehyde (30 min, RT), treated with 0.1% Nonidet P-40 (10 min, RT) and blocked with 2% bovine serum albumin (30 min, RT). Then, cells were incubated overnight at 4 °C with the following primary antibodies: rabbit anti-collagen type I (ab 34710, 1:500, Abcam, Cambridge, UK), mouse anti-collagen type III (ab6310, 1:1000, Abcam), and rabbit anti-elastin (ab21610, 1:200, Abcam). After 2–3 rinses in PBS, cells were incubated for 30 min with secondary antibodies (DyLight 594 conjugated goat anti*-*mouse antibody, 1:50, Abcam or fluorescein (FITC-conjugated goat anti-rabbit antibody, 1:200, Jackson ImmunoResearch, West Grove, PA, USA). Finally, Samples were washed with PBS, stained with DAPI (4′-6-diamidino-2-phenylindole, Sigma-Aldrich, St. Louis, MO, USA), and imaged using a fluorescence microscope (Eclipse Ti-E, Nikon Cooperation) with CCD camera system (SPOT RT3, Diagnostic instruments Inc.). For quantitative analysis of elastin, type I and III collagen in Hs68 fibroblasts, we used ImageJ software to measure the fluorescence intensity in 10 images obtained with equal acquisition parameters.

### 4.10. Statistical Analysis

The experiments were repeated at least twice with similar results, and the values were expressed as means ± standard deviations. The statistical analysis was conducted with SPSS, Version 24.0 (IBN, New York, NY, USA). A paired samples *t*-test was used for the statistical analysis of differences between two groups. Two-way analysis of variance with Dunnett’s multiple comparison was used for statistical analysis of the differences among multiple groups. Differences were considered to be statistically significant at *p* < 0.05.

## 5. Conclusions

In summary, ADM-induced lipogenesis in Hs68 fibroblasts may inhibit the expression of adiponectin while exogenous adiponectin administration can partially overcome UVA-induced reduction in the cell adhesion strength of Hs68 fibroblasts via the activation of AdipoR1 and the suppression of EGF-R. Moreover, exogenous adiponectin administration indicated a limited degree of recovery of elastic modulus on UVA-irradiated Hs68 fibroblasts through the increase of type III collagen and elastin mRNA expression and the decrease of MMP-1 and MMP-3 mRNA expression. Taken together, this finding could be useful in prevention of UVA-induced photoaging, particularly in obese individuals who may have reduced adiponectin level in skin.

## Figures and Tables

**Table 1:** Primer sequences used in the real-time PCR experiment.

Primer	Sequence (Forward)	Sequence (Reverse)
GADPH	CATGAGAAGTATGACAACAGCCT	AGTCCTTCCACGATACCAAAGT
PPAR-γ	AGCCTGCGAAAGCCTTTTGGTG	GGCTTCACATTCAGCAAACCTGG
LPL	GGACTTGGAGATGTGGACCA	TGCTGCTTCTTTTGGCTCTG
FABP4	AAAGTCAAGAGCACCATAACC	TTCAATGCGAACTTCAGTCC
Adiponectin	TATCCCCAACATGCCCATTCG	TAGGCAAAGTAGTACAGCCCA
EGFR	TGGCATCTTTAAGGGCTCCA	TGGCTAGTCGGTGTAAACGT
Adipo-RI	ACACCCTTCTCTGAGCCTTC	GGTGTGAAAGTGGGCTGAAG
Adipo-RII	TGGAGCCCATTTTAGAGGCA	CACCGACCTTCCCATACCTT
MMP-1	CTGGCCACAACTGCCAAATG	CTGTCCCTGAACAGCCCAGTACTT
MMP-3	ATTCCATGGAGCCAGGCTTTC	CATTTGGGTCAAACTCCAACTGTG
COX-2	GCCCTTCACGTTATTGCAGATG	ATATGTTCTCCTGCCTACTGGAA
Collagen-I	AAAAGGAAGCTTGGTCCACT	GTGTGGAGAAAGGAGCAGAA
Collagen-III	GAAGGGCAGGGAACAACTTG	TTTGGCATGGTTCTGGCTTC
Elastin	GGCCATTCCTGGTGGAGTTCC	AACTGGCTTAAGAGGTTTGCCTCCA
